# Air Trapping Mechanism in Artificial Salvinia-Like Micro-Hairs Fabricated via Direct Laser Lithography

**DOI:** 10.3390/mi8120366

**Published:** 2017-12-20

**Authors:** Omar Tricinci, Tercio Terencio, Nicola M. Pugno, Francesco Greco, Barbara Mazzolai, Virgilio Mattoli

**Affiliations:** 1 Center for Micro-BioRobotics @SSSA, Istituto Italiano di Tecnologia, Viale Rinaldo Piaggio 34, 56025 Pontedera, Italy; tercio.terencio@iit.it (T.T.); francesco.greco@iit.it (F.G.); barbara.mazzolai@iit.it (B.M.); virgilio.mattoli@iit.it (V.M.); 2 Laboratory of Bio-Inspired and Graphene Nanomechanics, Department of Civil, Environmental and Mechanical Engineering, University of Trento, 38123 Trento, Italy; nicola.pugno@unitn.it; 3 School of Engineering and Materials Science, Queen Mary University of London, Mile End Road, London E1 4NS, UK; 4 Ket Labs, Edoardo Amaldi Foundation, Italian Space Agency, Via del Politecnico snc, Rome 00133, Italy; 5 Department of Life Science and Medical Bioscience, Graduate School of Advanced Science and Engineering, Waseda University, 2-2 Wakamatsu-cho, Shinjuku-ku, 169-8480 Tokyo, Japan

**Keywords:** biomimetics, Salvinia, hierarchical structures, hydrophobic surface, air trapping, 3D laser lithography

## Abstract

Salvinia leaves represent an extraordinary example of how nature found a strategy for the long term retainment of air, and thus oxygen, on a surface, the so-called ‘Salvinia effect’, thanks to the peculiar three-dimensional and hierarchical shape of the hairs covering the leaves. Here, starting from the natural model, we have microfabricated hairs inspired by those present on the *Salvinia molesta* leaves, by means of direct laser lithography. Artificial hairs, like their natural counterpart, are composed of a stalk and a crown-like head, and have been reproduced in the microscale since this ensures, if using a proper design, an air-retaining behavior even if the bulk structural material is hydrophilic. We have investigated the capability of air retainment inside the heads of the hairs that can last up to 100 h, demonstrating the stability of the phenomenon. For a given dimension of the head, the greater the number of filaments, the greater the amount of air that can be trapped inside the heads since the increase in the number of solid–air interfaces able to pin the liquid phase. For this reason, such type of pattern could be used for the fabrication of surfaces for controlled gas retainment and gas release in liquid phases. The range of applications would be quite large, including industrial, medical, and biological fields.

## 1. Introduction

In recent years, engineering has been constantly directing interest to the biomimetics paradigm for the solution of technological issues [1]. Plants and animals offer a great variety of functional surfaces from which they take inspiration for superficial patterning [2,3,4,5]. The remarkable characteristic of such surfaces is the deep connection between materials and morphology: with a relatively small number of structural materials, nature has been able to achieve very complex functionalities by exploiting proper modeling of surfaces. In the case of plants, the ‘lotus effect’ has been widely investigated for the fabrication of self-cleaning surfaces [6,7,8,9]. Another promising phenomenon related to the pattern morphology of the leaves is the so-called ‘Salvinia effect’ [10,11,12,13,14,15,16,17]: when submerged by water, this floating fern can retain a thin layer of air on its surface thanks to the presence of ‘eggbeater’ hairs that can pin water on the tip while trapping air at the bottom. In this way the plant can have a reservoir of oxygen when under the water which can also provide thermal insulation. Both these properties could have a practical application in several technological fields. In particular the reproduction of such phenomena could be used in cases where the retaining of a gaseous phase in a liquid one is needed like in the case of thermal insulation, biological in vitro applications or for drag reduction like in the case of artificial nanofur inspired by Salvinia and *Notonecta glauca* [18,19]. 

**Figure 1:**
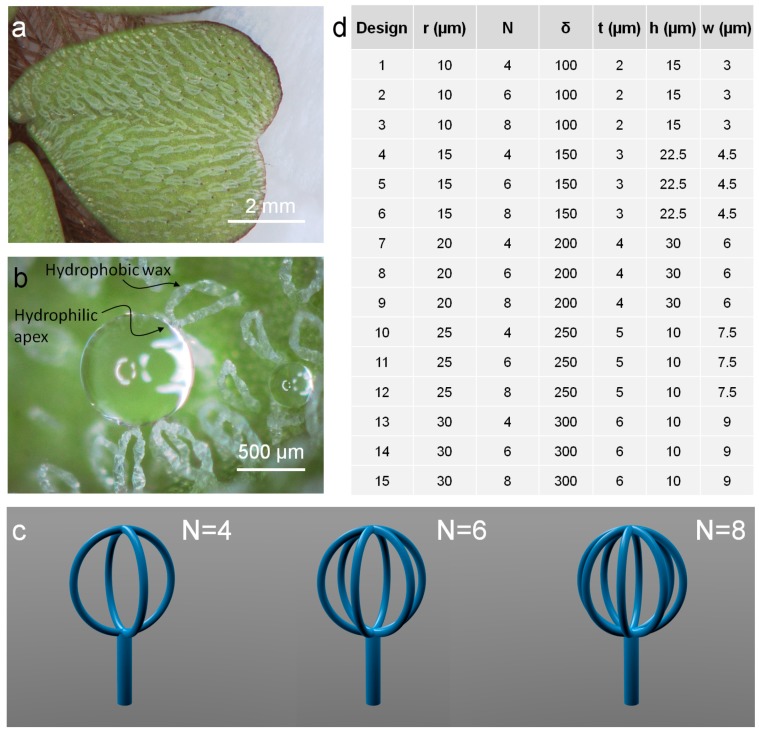
(**a**) Pattern of ‘eggbeater’ hairs that cover the upper side of the *Salvinia molesta* leaves; (**b**) Interaction of the hairs with a droplet water, showing the hydrophilic apex able to pin the droplet, while the remaining structure is hydrophobic; (**c**) Three different designs for the crown-like heads tested in our work consisting of four, six, and eight filaments obtained by the intersection of two, three, and four circumferences rotated by 90°, 60°, and 30° respectively; (**d**) Table with all the 15 designs studied for air retaining: *r* is the radius of the head, *N* the number of its filaments, *δ* is the number of parallel circumferences actually exposed to the laser beam for the fabrication of the filaments, *t* is their actual thickness, *h* is the height of the stalk, and *w* is its diameter.

In the case of *Salvinia molesta* (Figure 1a), the air retaining performance is due to the combination of complex three-dimensional patterning and chemical coating [10]. The hairs on the upper side of the leaf have a 1.5 mm long stalk which ends with a peculiar crown-like configuration made by four filaments, for a total height of the final quasi-spherical part of 500 µm. The apex of the filaments is composed of smooth dead cells, providing a sort of pinning point for the water droplets (Figure 1b), while the rest of the hair is covered by hydrophobic wax crystals. Exploiting this configuration, the plant is able to trap air both between the stalks and inside the crown-like heads. Here we investigate the latter aspect that is the dynamics and the persistence of the air inside the head of artificial hairs: how long the structures are able to retain air in the heads and how the shape and the volumes of such air bubbles change over time. 

Starting from the study of some peculiar plants and insects, the long term air retention mechanism has been investigated with the purpose of finding out the main features that a bioinspired surface should present in order to obtain the same performance. In fact, the main issue related to such surfaces is the instability of air layer due to external perturbations. The presence of hierarchical hairs and nanostructures, hydrophobicity, and micro- and nanocavities are all fundamental factors for reducing the instability of the air layer [9]. 

Moreover, Konrad et al. defined the mathematical model for the existence of the air–water interface and its relative mechanical stability and temporal persistence in presence of diffusion. We followed this model in order to perform the experiments in the same theoretical conditions [17]. 

How the geometrical disposition of the hairs affects the stability of the air layer has been already deeply investigated, even in the presence of hydrophilic structural materials that can show an opposite behavior—hydrophobicity—thanks to the three-dimensional arrangement, the microscopic morphology, and the roughness at the nano-scale [2,9]. For this reason, we downscaled the natural model from 8 up to 25 times, in the range of micrometers as characteristic dimension. As shown in our previous work, the dimensional scale and the peculiar features provide an air-retaining behavior without requiring chemical treatments [20]: in that study, we mainly focused on the study of hydrophobicity of the proposed microstructures built with an hydrophilic material, while in this paper we focus on the air trapping mechanism and dynamics of this phenomenon not yet investigated. Here we used a hydrophilic cross-linked epoxy-based photoresist for the micro-fabrication of artificial hairs by means of direct laser lithography. This technique is the only one that ensures outstanding results for 3D structures in terms of resolution at the microscale, with features of hundreds of nanometers [20,21,22]. In particular, we focus on two main aspects that can influence the dynamics and the time persistence of the air in the heads: the dimension and the geometry.

## 2. Materials and Methods

Salvinia-like artificial hairs were fabricated in negative tone SU-8 photoresist (MicroChem Corp., Westborough, MA, USA) on a glass substrate, by means of a direct laser lithography setup, Photonic Professional system (Nanoscribe GmbH, Eggenstein-Leopoldshafen, Germany). The laser paths coordinate corresponding to the desired shapes were programmed with MATLAB^®^ (R2016b, The Mathworks, Inc., Natick, MA, USA), allowing the fabrication of different designs. The preparation of the sample required several steps for ensuring the adhesion of the structures on the glass. First of all the glass substrate was rinsed with acetone, isopropyl alcohol (IPA), and deionized water. It was submerged for 10 min in a 50 mL ethanol solution with eight drops of 3-(Trimethoxysilyl) propyl methacrylate. The glass was rinsed with deionized water and dried with air. Then the photoresist was spin coated on the substrate at a speed of 1000 rpm in order to achieve a thickness of 100 µm, which is enough to contain the artificial hairs. After a pre-bake process (10 min at 65 °C followed by a second step at 95 °C for 30 min), the sample was mounted in the laser lithography system (Photonic Professional GT, Nanoscribe GmbH) and exposed to the laser beam with a center wavelength of 780 nm (Calman laser source). The writing speed was 25 μm·s^−1^ and the laser power 7.5 mW. After a post-bake phase (1 min at 65 °C followed by a second step at 95 °C for 10 min) the sample was developed for 20 min in SU-8 Developer (MicroChem Corp., Westborough, MA, USA) and rinsed with IPA and deionized water. 

The air trapping test was performed by submerging the sample with the microfabricated structures in water: in order to detect the presence of the air and to define the profile of the relative interfaces between structures, air and water, a dedicated setup based on a confocal microscope (C2 Confocal Microscope System, Nikon, Tokyo, Japan) was implemented. Since the SU-8 photoresist is fluorescent if excited at 401 nm, by marking the water with a fluorescent probe having different excitation wavelength, it is possible to selectively detect the structures, the water, the air, and their relative interfaces. For this reason, tetramethylrhodamine-5-(and-6)-isothiocyanate (5(6)-TRITC) (Life Technologies, Carlsbad, CA, USA) with a concentration of 0.01 mg·mL^−1^ was added to the water. Exciting the sample under the confocal microscope at 401 nm (excitation wavelength of SU-8) and 561 nm (excitation wavelength of TRITC), it was possible to distinguish them. Since experiments required prolonged immersion of the structures in small volume of liquid, samples were encapsulated by means of a poly-(dimethylsiloxane) (PDMS, Sylgard 184, Dow Corning Corp., Midland, MI, USA) ring (internal diameter 8 mm; thickness 200 μm) placed on the substrate glass around the structures and sealed with an additional glass after the deposition of 10 μL of TRITC-marked water.

The analysis of the air-retention performance of the samples was carried out by recording images of the microstructures (hairs) at different times; in particular, stacks of the crown-like air were acquired in order to obtain slices at a vertical distance of 500 nm. The scanning time for each slice is 4.6 s. According to the dimensions of the heads (related to the number of slices), the volumetric reconstruction has required a time between 3 min and 9 min.

It was possible to reconstruct the volume of the air inside the heads, finding out how the air–water interface changed over time, quantitatively evaluating the performance of different designs of the hairs. The detection of the interfaces in each image and the calculation of the air volumes were performed with a dedicated image processing script in MATLAB^®^. Air volumes were estimated by adding the air area in each slide multiplied by the vertical distance.

Static contact angle measurements were performed on flat SU-8 (100 μm thick) on glass, by means of an optical tensiometer (Attension, Biolin Scientific, Gothenburg, Sweden). Droplets of 5 μL water and water with 5(6)-TRITC (0.01 mg·mL^−1^) were tested in order to verify the effect of the colorant on the wettability properties.

All SEM images of the artificial hairs have been acquired with an EVO MA10 (Zeiss, Oberkochen, Germany). 

## 3. Microfabrication Results

The design of the artificial hairs has followed the main specifications for ensuring the presence of a trapped air layer [9]. The two main geometrical parameters investigated in the air-retention mechanism were the dimension of the crown-like heads and their shape. Five different radii of the heads were tested, from 10 µm to 30 µm with increments of 5 µm. Although the hairs of the *Salvinia molesta* have only four filaments in the apical part, we also investigated the possibility of changing their number and to study the effect of such modification of the number of solid–air–liquid interfaces. Hairs with four (like in the real plant), six, and eight filaments were implemented (Figure 1c). The first head design was obtained by intersecting two circumferences at 90°, the second three circumferences at 60°, and the last four circumferences at 30°. Also the thickness of the filaments has been tuned according to the dimension of the heads, from 2 µm to 6 µm with increments of 1 µm. Hairs with a diameter of the head of 50 µm and 60 µm were fabricated with a shorter stalk in order to remain inside the 100 µm thickness of the spin coated photoresist. All salvinia-like artificial hairs were fabricated in a 10 × 10 array configuration, for a total of 15 different designs, as summarized in Figure 1d. 

**Figure 2:**
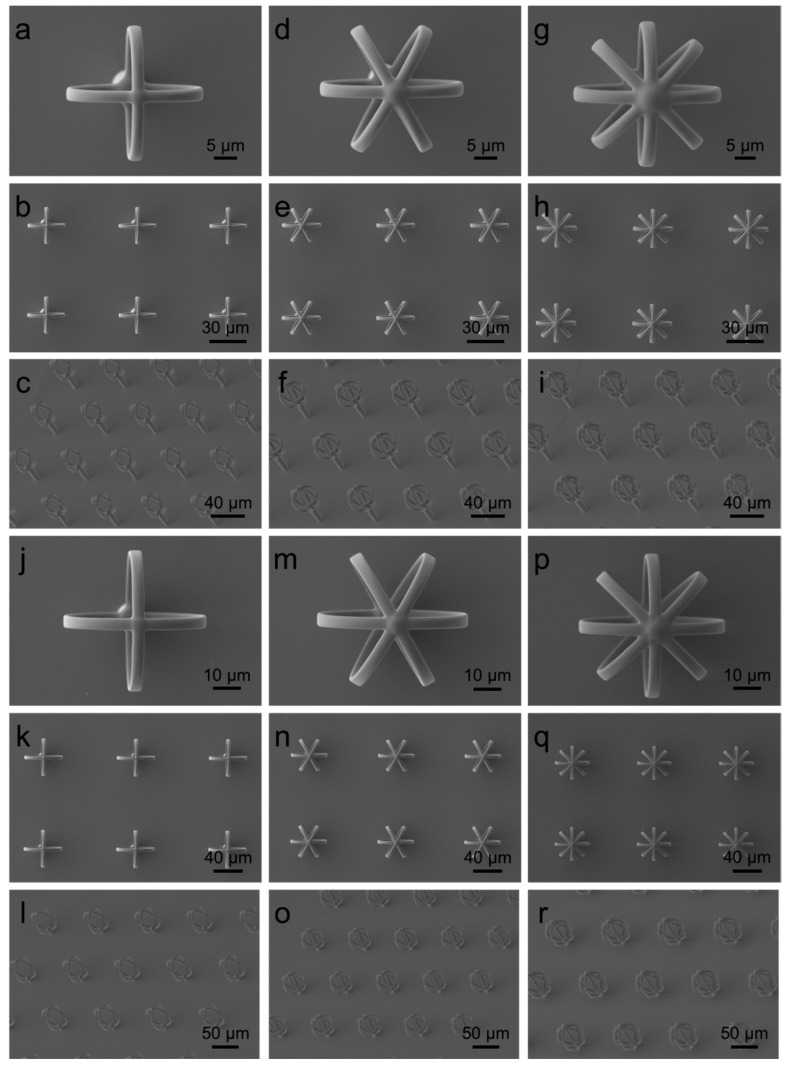
SEM images of the results of the microfabrication of some hairs designs by means of direct laser lithography: hairs with head radius of 15 µm and four (**a**–**c**), six (**d**–**f**), and eight (**g**–**i**) filaments (design number 4, 5 and 6 respectively). Hairs with head radius of 25 µm and four (**j**–**l**), six (**m**–**o**) and eight (**p**–**r**) filaments (design number 10, 11 and 12 respectively). See Figure 1d for all the geometrical parameters of the presented designs.

In Figure 2, the main microfabrication results for hairs with heads with radius of 15 µm and 25 µm are illustrated. All the SEM images of the designs are shown in Figure S1. The hairs were reproduced with outstanding results in terms of resolution: the heads in particular are characterized by smooth filaments (visible in the details of the single heads in Figure 2) due to the fact that the resist was exposed to the laser according to a real three-dimensional path and not in a slice-by-slice fashion like in other lithographic techniques.

## 4. Experimental Results and Discussion

Since for all the experiments 5(6)-TRITC has been added to the water, contact angle measurements were performed in order to investigate the effect of the colorant on the wettability characteristics of the microstructures. As reported in Figure S2, the contact angles of water and water with 5(6)-TRITC were 59° ± 0.4° and 58° ± 0.3°, demonstrating the negligible effect of the colorant presence.

According to Konrad et al. [17], an air bubble submersed in water can persist only if its pressure (*p_a_*) is lower or equal the above atmospheric pressure (*p*_atm_). In fact, when *p_a_* > *p*_atm_, air particles start diffusing from the bubble and reach the atmosphere. 

Considering an air bubble in water, starting from Young–Laplace Equation [17],

(1) $$p_{a}=p_{w}+\frac{2\sigma }{R}$$

(where *p_w_* is the water pressure at the air–water interface, *σ* is the surface tension, and *R* is the radius of the bubble), and considering that *p_w_* depends on the submersion depth *h* [17],

(2) $$p_{w}=p_{atm}+\rho_{w}gh$$

(where $\rho_{w}$ is the water density and *g* the gravitational acceleration), it is possible to find, in case of existence, the maximum depth for air bubbles persistence [17]

(3) $$h<\frac{2\sigma }{\rho_{w}gR}$$

Replacing the relative values for *σ* (0.073 N/m), *R* (30 µm, the worst case), $\rho_{w}$ (10^3^ kg/m^3^), we obtain a limit depth *h* of about 50 cm which is fully satisfied in our experiments. 

**Figure 3:**
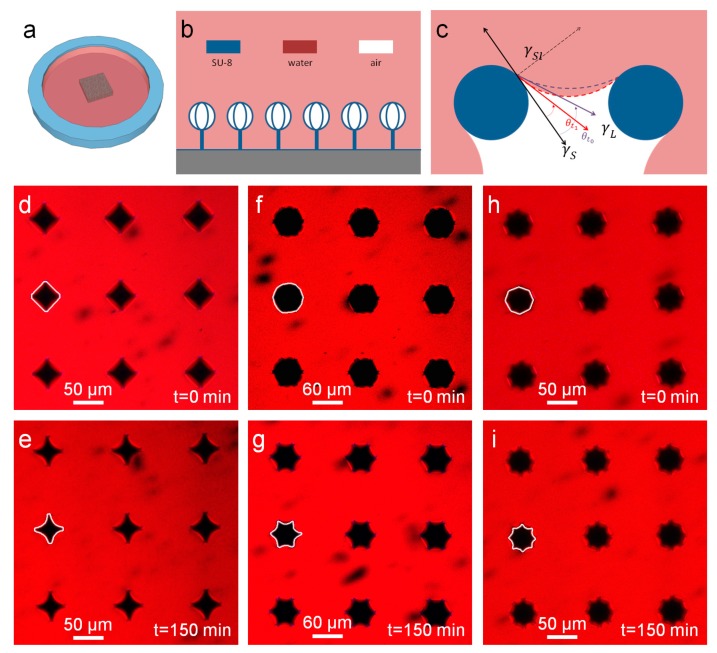
(**a**) Sketch of the sample used for the experimental procedure at the confocal microscope: the array (the square in the center) with the structures is confined by a poly-(dimethylsiloxane) (PDMS) ring (in blue), filled with water marked with tetramethylrhodamine-5-(and-6)-isothiocyanate (5(6)-TRITC) (in red) and then sealed with a thin glass slide on the top (not shown); (**b**) Scheme of the ‘macro’ Wenzel state (the water fills all the room over the hairs and between the stalks) and the ‘micro’ Cassie–Baxter (the water cannot reach the inside of the heads), that are experienced by all the tested samples; (**c**) Scheme (not in scale) of the interaction of water (in red), air (in white) and SU-8 (structural material, in blue), related to Equation (1). The red and violet arrows show the variation of the contact angle *θ* during time (from *θ*_t0_ to *θ*_t1_ respectively); (**d**) Example of heads with four filaments (design 10) recorded at the confocal microscope, showing the profile of the air–water interface (in white) at the starting point of the experiment; (**e**) Same sample reported in (**d**) after 150 min, showing the variation of the air–water interface (in white). Similarly, in (**f**,**g**) examples of heads with six filaments (design 14) and in (**h**,**i**) of eight filaments (design 12), taken at the starting point of the experiment and after 150 min.

In fact, whatever is the tested design, once the artificial hairs are submerged in the water and sealed (Figure 3a), the water forms a layer and fills the room between the structures, leaving the air inside the crown-like heads. This evidence suggests a first consideration about the dynamics of the interaction of the materials involved in the process. The hairs patterned on the glass represent a non-ideal rough solid surface for the water whose behavior can be described in terms of two different models, Wenzel and Cassie−Baxter, that are usually used for the computation of the contact angles between the phases [8,11,23,24]. In the proposed geometry, there are two states that can exist at the same time: a ‘macro’ Wenzel state in which the water fills all the room over the hairs and between the stalks, and a ‘micro’ Cassie–Baxter state corresponding to the crown-like heads [20], as illustrated in Figure 3b. The estimation of the contact angle is not possible in the proposed design for the Wenzel phase since the water does not assume the shape of a droplet, but only for the Cassie–Baxter phase in the heads. In fact, the static contact angle *θ* (Figure 3c) can be approximately estimated by adapting the Young equation [25] that explains the relationship between the surface free energy of the solid *γ_S_*, the liquid surface tension *γ_L_* and the solid-water interfacial energy *γ_SL_*

(4) $$\gamma_{L}\cos\theta =\gamma_{S}-\gamma_{SL}$$

Anyway, the value of the contact angle and thus the shape of the profile of the water–air interface changes over time, due to the variation of the air volume inside the heads till it reaches a stable (or metastable) configuration. In fact, it is important to underline that for the micrometer-sized textured considered, the free-energy barriers are expected to be so large that metastable states can be very long-lived [13,26]. While the air diffuses into the water, the decrease of the contact angle is caused by the protrusion of the water inside the crown-like heads (Figure 3c), making the shape of the air bubble more and more different to that of a sphere. At steady state, the curvature of the liquid–vapor interface will depend from the difference of pressure (inside/outside the bubble) following the Young–Laplace Equation (4).

All the tested designs experienced the same combination of ‘macro’ Wenzel and ‘micro’ Cassie–Baxter states. The first one is due to the fact that the Cassie–Baxter state is a higher energy state and so disadvantaged from a thermodynamic point of view. The energy barrier needed for the switching between the two states is proportional to the number of structures for unity of area [18]: in the proposed samples the density of the structures is not enough for ensuring the Cassie–Baxter state between the stalks, thus isolating the phenomenon of air trapping inside the crown-like heads. In this regard, it is important to underline that, as reported in a previous work [20], an additional mixed state, *“*macro-Cassie−Baxter and micro-Wenzel”, can be compatible with proposed equations. Nevertheless, we do not observed it in any combination of geometrical parameters for our basic shape. We speculate that, in order to obtain this state, a combination of materials with different surface energy (with proper design) could be necessary.

The analysis at the confocal microscope confirms the presence of ‘macro’ Wenzel and ‘micro’ Cassie–Baxter states as illustrated in Figure 3d–i, where it is represented, as an example, the middle section of the heads. In Figure 3d,f,g three different geometries for the heads (*N* = 4, 6, 8) show the structures at the beginning of the experiment: at this moment, the volume of air inside the heads assumes a peculiar shape in each design, in particular the higher the number of filaments, the more similar to a sphere the shape is. This means that four filaments can trap the lowest amount of air compared with the other geometries: the reason is in the reduced number of solid interfaces able to pin the water. As time goes by, the dimension of the air volume decreases as shown in Figure 3e,g,i, which are the corresponding images taken after 150 min; after this change that happens in the first minutes, the shape of the water–air interface seems to settle to a definitive final conformation, proving also the stability of the system at least in static conditions.

**Figure 4:**
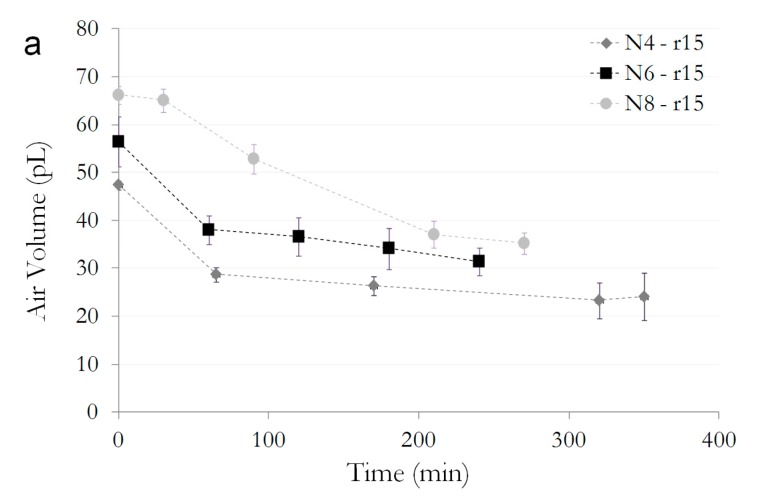

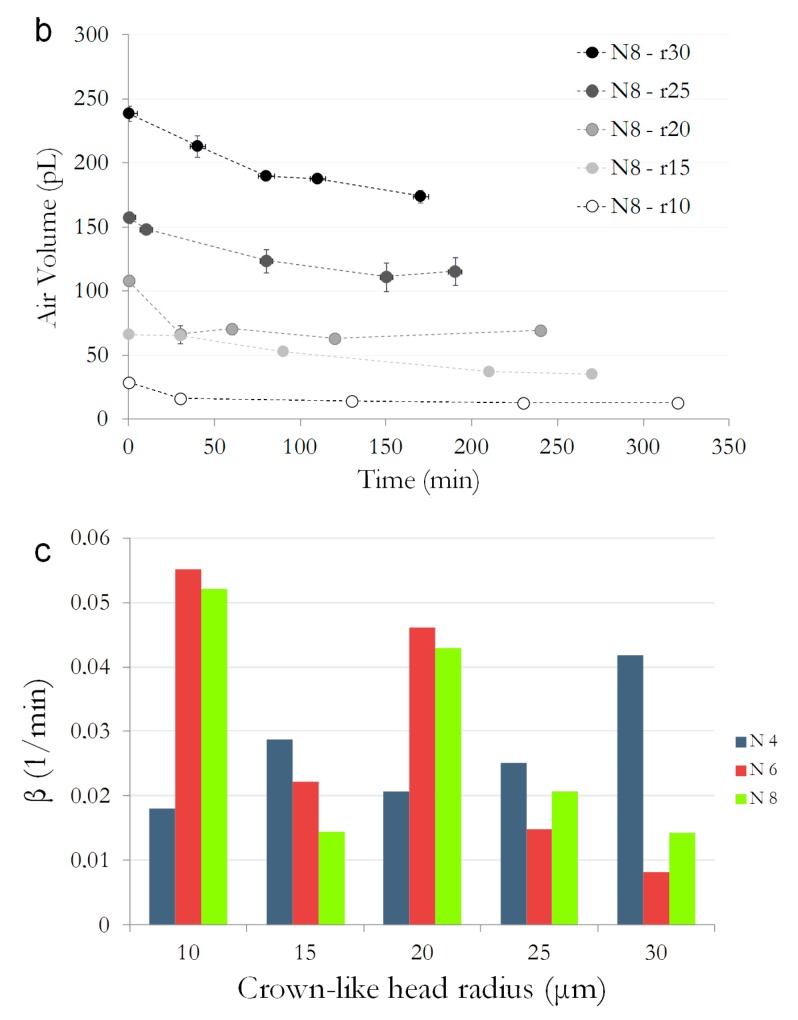
(**a**) Trend of the air volume trapped inside the crown-like heads with a radius of 15 μm for different numbers of filaments; (**b**) Variation over time of the volume of air retained inside the crown-like heads in the case of *N* = 8, for different values of the radius;(**c**) Comparison, for all designs, of the coefficients *β* related to the rate of reduction of air inside the heads.

In order to quantitatively estimate the volumes of air trapped inside the crown-like heads, for each design the sum of the section areas has been calculated by means of a MATLAB^®^ program, starting from the pictures acquired with the experimental procedure already described. In Figure 4, there are the results of the dynamics of the variation of the air volumes during time. Setting the dimension of the radius of the heads and comparing the different number of filaments, it is evident what was qualitatively appreciable from Figure 3d,f,g, that is a higher number of filaments is able to trap more air (as in the example of radius 15 µm in Figure 4a). On the other hand, setting the number of filaments and varying the dimension of the heads, the bigger one can trap, quite obviously, bigger air volumes (as in the example of *N* = 8 in Figure 4b). The results of dynamics of the variation of the air volumes trapped in the salvinia-like structures for all the designs in Figure 1d are illustrated in Figures S3 and S4.

To quantitatively evaluate the dynamics of volume reduction, we approximated that variation with a descending exponential trend, compatibly with a first order kinetics, as

(5) $$V=\left(V_{i}-V_{f}\right)e^{-\beta t}+V_{f}$$

where *V_f_* is the final volume of air (stable persistent value), *V_i_* is the value at the starting point and *β* (min^−1^) is a coefficient associated with the rate of the variation of the volume. The fluctuation of the volume until a stable value inside the heads is reached could be due to effects of gravity (even if it is expected to play a minor role at that scales) and pressure fluctuations, but mainly to air diffusion in the water since the sample containing the structures is completely sealed and there is no way for the air to reach the external environment (PDMS is somehow permeable to air, but, due to geometry of the sample, this effect is neglegible). This aspect is also confirmed by the fact that the Cassie–Baxter configuration remains stable up to 100 h. Moreover, it is important to underline that air diffusion in liquid is low dynamics which, however, does not alter the long-term thermodynamics [27]. In respect, the final micro Cassie–Baxter state might indeed be metastable also in an open system if the structure (as in our case) owing to the re-entrant filaments. Nevertheless, this aspect was not investigated in open configuration because evaporation limits the sample living time.

Fitting the experimental data with the model Equation (5), we have extrapolated and compared the values of the parameter *β* of the various designs as shown in Figure 4d. Values for *β* of the same order of magnitude can be deduced from the data, thus meaning that there are not really significant differences, at least at this dimensional scale, for the rate of air release from the crown-like hairs: it is practically of the same magnitude for all the designs, showing that it is quite likely related to the intrinsic mechanism of diffusion of the air inside the water. For this reason, the shape and the dimension could be simply chosen for having the bigger possible amount of air inside the crown-like heads, that is: a high number of filaments and, of course, big radii of the heads. Filaments also ensure another property related to the structural stability of the hairs that result in more resistance to mechanical stress.

## 5. Conclusions

In this work, we have implemented a procedure for the fabrication of artificial hairs in the microscale, inspired by those present on the upper side of the *Salvinia molesta* leaf, that have the peculiar characteristic of trapping air inside their crown-like heads. The structures have been made by means of direct laser lithography, which allows for outstanding results in terms of resolution in the micrometer and nanometer scale, thus leading to the fabrication of robust hierarchical microstructures. The hairs have been dimensionally scaled down to 8–25 times respect to the natural ones and have been fabricated in hydrophilic cross-linked epoxy-based photoresist whose wettability has been turned into ‘air-retaining’ thanks to the proper design of the crown-like heads morphology, without requiring any chemical coating. By changing the dimension of the heads and the number of filaments composing them, it has been possible to determine the best configuration in order to retain a greater amount of air: larger radii and higher number of filaments. The artificial structures have demonstrated their performance in long term air retention up to 100 h. The amount of air inside the heads changes during time, following an exponential trend whose rate seems to be not strongly dependent from the tested morphologies, at least at the micrometric scale.

This type of structure could have a great technological applications in several fields where the retention of oxygen or other gasses is needed, or for thermal insulation applications. The proposed study is the first step in the study of this phenomenon and could be extended to even greater proportions, where proper surface patterning could be made on larger production scales.

## Supplementary Materials

The following are available online at www.mdpi.com/2072-666X/8/12/366/s1, Figure S1: SEM images of design 1 (a,b); 2 (c,d); 3(e,f); 7 (g,h); 8 (i,j); 9 (k,l); 13 (m,n); 14 (o,p); 15 (q,r). Designs refers to table in Figure 1d, Figure S2: (a) Static contact angle of water on flat SU-8; (b) Static contact angle of water with 5(6)-TRITC (0.01 mg·mL^−1^) on flat SU-8, Figure S3: (a–e) Results of the dynamics of the variation of the air volumes trapped in the salvinia-like structures for all the tested designs (see Figure 1d), grouped for crown-like radius heads. *N* represents the number of filaments composing the crown-like heads while *R* is the radius of the heads, Figure S4: (a–c) Results of the dynamics of the variation of the air volumes trapped in the salvinia-like structures for all the tested designs (see Figure 1d), grouped for number of crown-like heads filaments. *N* represents the number of filaments composing the crown-like heads while *R* is the radius of the heads.
