# Supplementary material for: Air Trapping Mechanism in Artificial Salvinia-Like Micro-Hairs Fabricated via Direct Laser Lithography
Source: Micromachines (Basel). 2017 Dec 20;8(12):366. doi: 10.3390/mi8120366 (PMC6187866; doi:10.3390/mi8120366)
Supplement: Supplementary file 1 [file micromachines-08-00366-s001.pdf]

Supplementary Materials: Air trapping mechanism in artificial Salvinia-like micro-hairs fabricated via direct laser lithography

Omar Tricinci, Tercio Terencio, Nicola M. Pugno, Francesco Greco, Barbara Mazzolai and Virgilio Mattoli

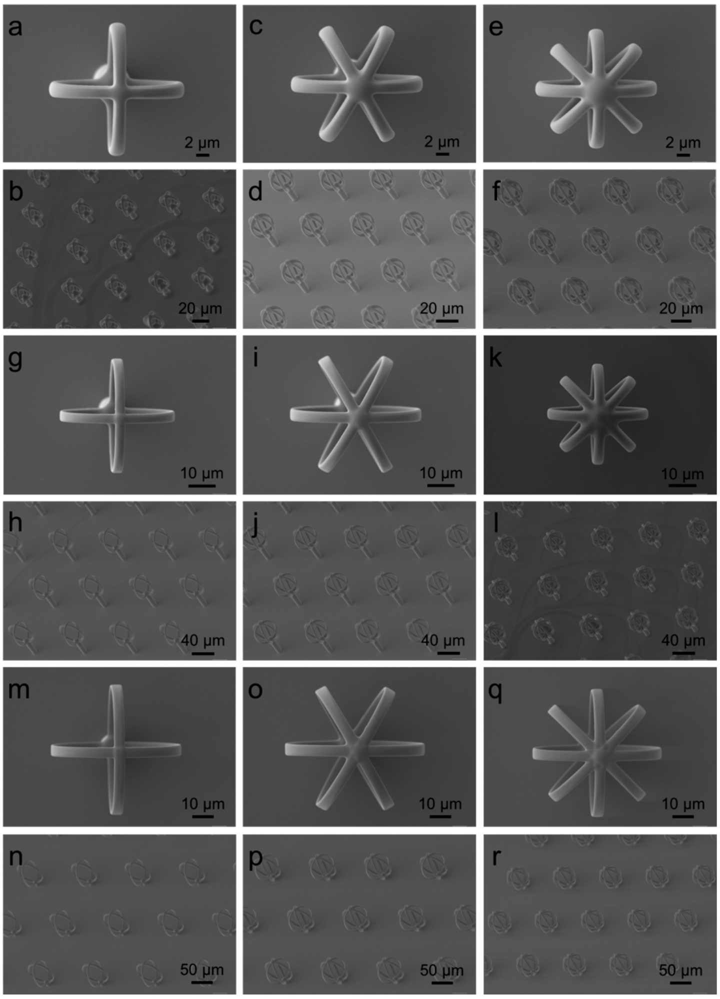

Figure S1. SEM images of design 1 (**a**,**b**); 2 (**c**,**d**); 3(**e**,**f**); 7 (**g**,**h**); 8 (**i**,**j**); 9 (**k**,**l**); 13 (**m**,**n**); 14 (**o**,**p**); 15 (**q**,**r**). Designs refers to table in Figure 1d.

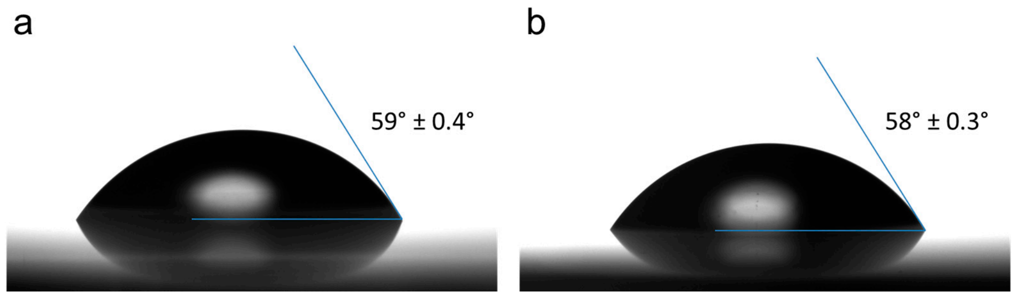

Figure S2. (**a**) Static contact angle of water on flat SU-8; (**b**) Static contact angle of water with 5(6)-TRITC (0.01 mg·mL^{-1}) on flat SU-8.

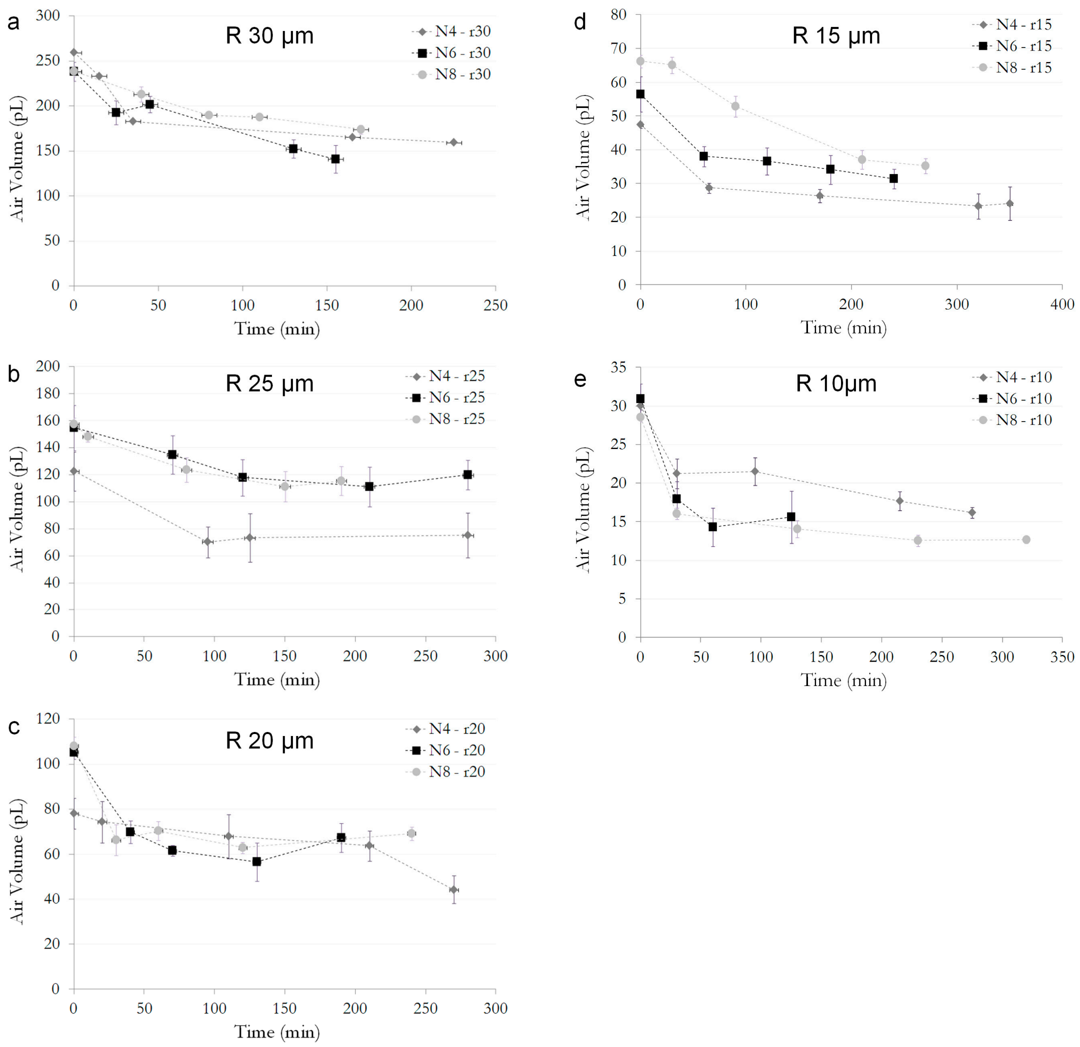

Figure S3. (**a**–**e**) Results of the dynamics of the variation of the air volumes trapped in the salvinia-like structures for all the tested designs (see Figure 1d), grouped for crown-like radius heads. *N* represents the number of filaments composing the crown-like heads while *R* is the radius of the heads.

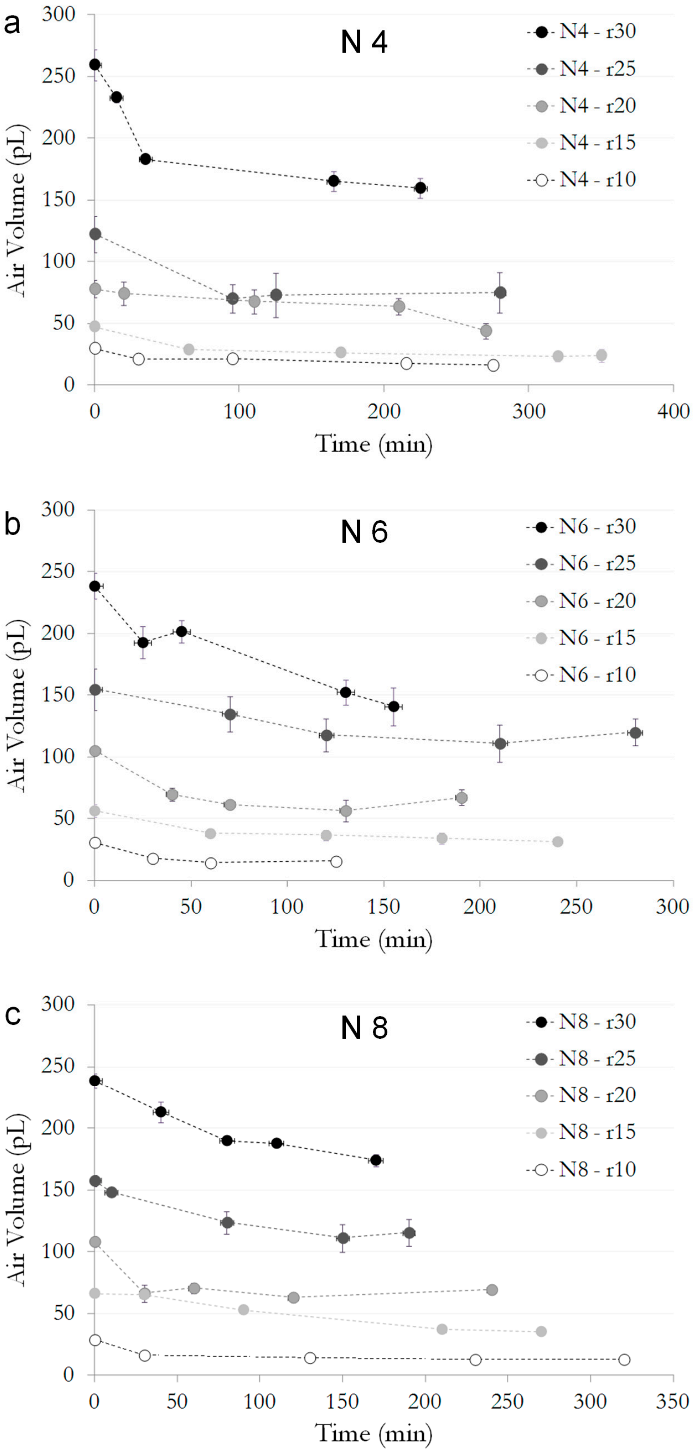

Figure S4. (**a**–**c**) Results of the dynamics of the variation of the air volumes trapped in the salvinia-like structures for all the tested designs (see Figure 1d), grouped for number of crown-like heads filaments. *N* represents the number of filaments composing the crown-like heads while *R* is the radius of the heads.
